# Giant Dedifferentiated Gastric Liposarcoma: Largest to Date

**DOI:** 10.7759/cureus.39595

**Published:** 2023-05-28

**Authors:** William G Baker, Shayla Albright, Tiffany Aragan, Raul Mederos

**Affiliations:** 1 Surgery, American University of Antigua, New York, USA; 2 General Surgery, Hialeah Hospital, Hialeah, USA

**Keywords:** cdk4, mdm2, gastrectomy, dedifferentiated liposarcoma (ddlps), gastric, liposarcoma

## Abstract

Liposarcoma is one of the most common soft tissue malignancies in adults, accounting for 15% to 20% of all sarcomas. We report a case of the largest dedifferentiated gastric liposarcoma recorded to date in a patient who presented with upper gastrointestinal bleeding. Initial pathology reports indicated a benign cause even after multiple biopsies were performed; only after surgical resection was the diagnosis confirmed. We discuss histopathology, genetic markers, and differential diagnoses.

## Introduction

Liposarcoma is the most common adult malignancy of soft tissues and is mostly known to be present in the retroperitoneal area and extremities, depending on subtype classification. Well-differentiated liposarcoma (WDLPS) and dedifferentiated liposarcoma (DDLPS) subtypes have been found in the retroperitoneum, while myxoid and pleomorphic subtypes are typically found within the extremities [1]. It is often misdiagnosed due to its rarity and vague symptoms. Diagnosis is usually confirmed after a pathological examination of the surgical specimen. Standard superficial biopsies typically are of little yield due to the submucosal origin of the tumor [2]. Only 39 previous studies have shown liposarcoma in the upper gastrointestinal system with a mean age of 57.0 years at the time of diagnosis with varying tumor sizes ranging from 0.5 cm to 36 cm [3]. Of the 39 previous gastric liposarcomas documented, two are dedifferentiated (5.1%), 17 are well-differentiated (43.6%), four are mixed (10.3%), nine are myxoid (23.1%), three are pleomorphic (7.7%), and four are unknown (10.3%) [3]. Of the two DDLPS that have been reported, one was reported as being 1 cm and the other is of an unknown size [3]. DDLPS are a high-grade neoplasm consisting of areas of non-lipogenic sarcomatous tissue and may have previously been a WDLPS that has since progressed [4]. Symptoms may include abdominal pain/discomfort, dark stools, generalized weakness, anemia, nausea, anorexia, dyspepsia, etc. Treatment for gastric liposarcomas has been surgical resection with evidence that chemotherapy and/or radiation has shown to not be of benefit in the limited research [3].

It is known that tumor markers are important for the diagnosis of different tissue origins, and differentiation of various liposarcomas is no exception. Previous gastric liposarcoma reports have shown tumor markers positive for S100, CD34, SMA, Desmin, MDM2, and CDK4 [1,3]. Murine double minute 2 homolog (MDM2) gene overexpression and CDK4 gene amplification have been found to be prevalent in soft tissue sarcomas and well-differentiated/DDLPS, respectively [5]. Here, we present a case of a large dedifferentiated gastric liposarcoma that happened to be larger than imaging previously predicted.

## Case presentation

**Figure 1:**
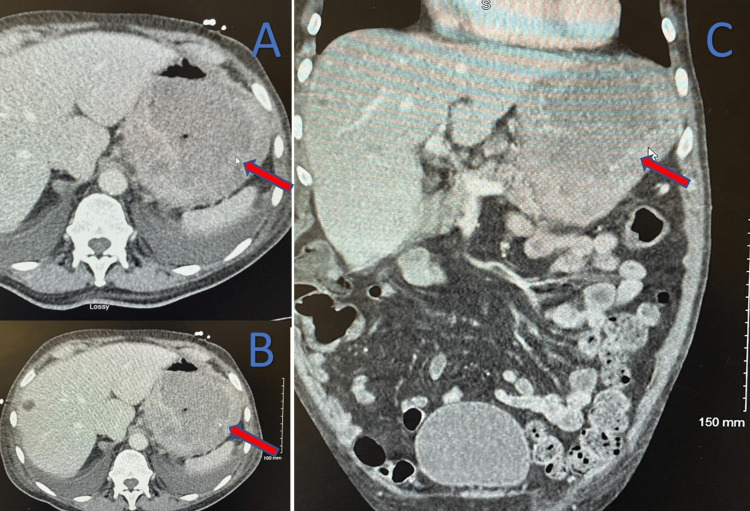
CT showing thickened gastric mucosa (red arrows). A and B axial images. C coronal image.

A 71-year-old man with a past medical history of blindness and retinitis pigmentosa presented to the emergency department with symptoms of generalized weakness for several days along with dark/black stools. His hemoglobin was found to be 5.0 g/dL with a leukocytosis and elevated troponins likely secondary to severe anemia [6]. The patient was then admitted and the physical exam was unremarkable. The patient’s vital signs showed a heart rate of 70 bpm, temperature of 97.6 F, respiratory rate of 18 breaths per minute, blood pressure of 118/66 mmHg, and oxygen saturation of 99%. The patient was transfused with blood products, and his hemoglobin improved to 7.6 g/dL. Radiologic images were collected to better determine the etiology. A contrast abdominal and pelvic CT showed fecal impaction and abnormal gastric mucosal thickening with enhancement suspicious of malignancy (Figure 1). No hepatic metastases were seen. Due to these abnormal results, an upper endoscopy was performed which showed a large ulcerated fundic mass which was then biopsied. Multiple biopsies were obtained, but due to their superficial depth, only nonspecific inflammation of gastric mucosa was seen which is a common theme for gastric liposarcomas [7]. Pathologic reports returned as a possible benign gastric xanthoma. Surgery was not indicated as it appeared to be a benign condition, and the patient elected not to have a surgical biopsy. The patient was later discharged home with a scheduled follow-up.

The patient returned to the emergency department three weeks later with recurrent complaints of dark/black stools. A second upper endoscopy was performed once again showing a 10 cm irregular friable mass extending from the cardia to the lesser curvature. Deeper biopsies were taken and sent off for consultation which returned as a DDLPS negative for *H. pylori*. Tumor cells were reported as S100+, C-KIT+, CD68+, and HMB-45+ while negative for CD45, CD34, and cytokeratin AE1/AE3.

**Figure 2:**
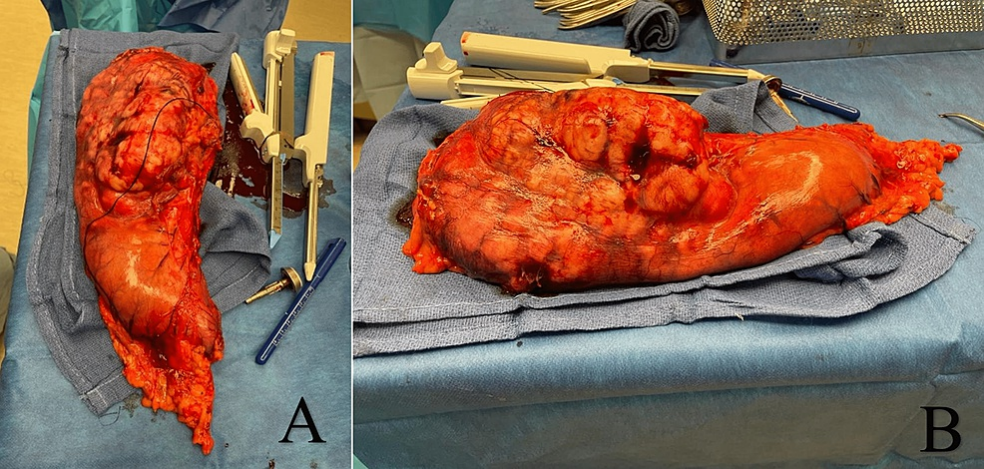
A and B showing gross specimen.

The following day, surgical consultation was done, and a proposed exploratory laparotomy was planned. During the procedure, there was extensive tumor involvement in the stomach, and a decision was made to proceed to a total gastrectomy with Roux-en-Y esophagojejunostomy reconstruction under general anesthesia. Partial resection was impossible due to the tumor invasion into the stomach. A gastric mass was found in the fundus and body of the stomach. The tumor appeared contained within the stomach with some lymphadenopathies noted at the gastroesophageal junction. A D1 lymphadenectomy was then performed. No liver lesions were identified, and the remainder of the intestines were grossly unremarkable. The patient tolerated the procedure well without any complications. The gross specimens were sent to pathology. Pathologic surgical report showed a transmural DDLPS measuring 18 cm x 13.5 cm x 3 cm. The tumor involved almost the entire posterior gastric wall, partially the lesser curvature with extension into the perigastric adipose tissue with lymphovascular invasion (Figure 2). Histologic grade by the French Federation of Cancer Centers Sarcoma Group Grade 2 and pathological staging (pTNM) primary tumor: pT2. Distal and proximal sections of the surgical specimen were free of malignancy. A postoperative upper GI X-ray kidney urinary and bladder with contrast was performed to assess the postoperative gastrectomy which found no obstruction or extravasation of the oral contrast, indicating no anastomotic leak.

The patient was sent home 14 days postoperatively and was also referred to gastroenterology for post-gastrectomy care with proper nutritional counseling and follow-up. The patient did not undergo any adjuvant treatment and will be followed by his primary care provider.

## Discussion

Liposarcomas are the most common soft tissue sarcomas arising in adults with a peak incidence between the age of 50 and 65 years old [1,7]. Liposarcomas comprise approximately 20% of all malignant mesenchymal neoplasms [8]. Histologically, liposarcomas are classified into five subtypes: well-differentiated, dedifferentiated, myxoid, round cell, and pleomorphic [9]. Among the different pathologically defined variants of liposarcoma, WDLPS are the most common, followed by DDLPS: both subtypes have been found in the retroperitoneum [1]. The remaining liposarcoma subtypes including myxoid, round cell, and pleomorphic liposarcomas are uncommonly found in the retroperitoneum [1].

Gastric liposarcoma originates as a direct result of the proliferation of undifferentiated mesenchymal cells and lipoblasts present in the gastric submucosa and muscular layer [2,10]. These gastric masses are commonly located at the lesser curvature or the antrum [10,11]. The overall mortality of liposarcoma is dependent on location and histological subtype, ranging from zero for atypical lipomatous tumors of the extremities to nearly 80% for tumors located in the viscera and retroperitoneum [7,12].

Often misdiagnosed due to their vague symptomatology and infrequent occurrence, definitive diagnosis of liposarcoma subtypes is, therefore, confirmed by pathological examination post-excision of the resected tissue mass. However, preoperative diagnosis has proven challenging due to the diagnostic limitations of endoscopy for gastric submucosal tumors, as was initially seen in our patient. In addition to the performance of a CT as indicated, the endoscopic ultrasound has been noted as the most effective diagnostic tool in both identifying such neoplasms arising from the submucosa and excluding other diagnoses [7]. Endoscopic ultrasound was not performed in this case since the presumed definitive diagnosis was a benign gastric xanthoma on superficial biopsies, though it was warranted.

Differential diagnosis of gastric liposarcoma tumors includes gastric stromal tumors, the two most common primary non-epithelial neoplasms of the stomach, lymphomas, peritoneal carcinomatosis, peritoneal liposarcoma, carcinoma engulfing perivisceral fat, hepatic metastasis adjacent to the stomach, and primary tumor of the omentum [7,12].

Treatment of choice is wide en-bloc surgical resection with clear, sufficient resection margins to decrease the possibility of malignancy [12,13]. In such case that a tumor arises near the cardia, the indicated resection involves a proximal gastrectomy with resection of the distal esophagus generally followed by reconstruction with esophagogastrostomy [12]. This mechanism, however, usually leads to severe gastroesophageal reflux; thus, a proximal gastrectomy with Roux-en-Y esophagojejunostomy whereby leaving the antrum in place can alternatively be done by inhibiting the reflux byproduct [13].

Upon exploratory laparotomy of our patient, the transmural DDLPS/gastric mass located in the body and fundus was found measuring 18 cm x 13.5 cm x 3 cm involving nearly the entire posterior gastric wall and partially the lesser curvature with extension into the perigastric adipose tissue with lymphovascular invasion. Since the tumor was so large, a partial gastrectomy was impossible; therefore, total gastrectomy with Roux-en-Y esophagojejunostomy reconstruction was performed in this particular case.

DDLPS are known to be characterized by the presence of sharply demarcated regions of non-lipogenic sarcomatous tissue within a well-differentiated tumor [14]. It is postulated that DDLPS may have formerly been WDLPS that have progressed due to their shared tumor markers as positive for S100, CD34, SMA, Desmin, MDM2, CDK4, as well as the overexpression of the MDM2 homolog gene and CDK4 gene amplification [1,3-5].

To date, only 39 previous studies have reported liposarcoma found in the upper gastrointestinal system with tumor sizes ranging from 0.5 cm to 36 cm [3]. In examining the 39 reported cases, only two of the 39 cases of gastric liposarcoma medically documented have been pathologically confirmed as the DDLPS subtype. Of the two DDLPS reported, one was recorded as being 1 cm in size, and the other was reported as of unknown size [3].

Therefore, we may definitively assume that this case documents the largest dedifferentiated gastric liposarcoma recorded to date. This may be the third-ever reported, as well as the largest reported case of a dedifferentiated gastric liposarcoma on medical record. Due to a gastric liposarcoma’s submucosal origin, recurrent negative or benign biopsies should prompt the clinician to include liposarcoma in the differential diagnosis of an upper gastrointestinal bleed [7], especially when radiological images point to a more malignant cause or underlying pathology.

## Conclusions

Gastric liposarcomas are rare with vague symptoms typically presenting as an upper gastrointestinal bleed. Complications can be lethal such as hemorrhagic shock, life-threatening anemia, and metastasis. It is imperative to include liposarcoma in the differential diagnosis of upper gastrointestinal bleeding, especially if multiple biopsy reports return benign. More research is needed for earlier detection to prevent morbid complications. Possible improved surgical techniques and ultimately disease confirmation through a proposed deeper biopsy device on upper endoscopy could improve earlier and less invasive disease detection.
